# Voxel‐based supervised machine learning of peripheral zone prostate cancer using noncontrast multiparametric MRI

**DOI:** 10.1002/acm2.12992

**Published:** 2020-08-08

**Authors:** Neda Gholizadeh, John Simpson, Saadallah Ramadan, Jim Denham, Peter Lau, Sabbir Siddique, Jason Dowling, James Welsh, Stephan Chalup, Peter B. Greer

**Affiliations:** ^1^ School of Mathematical and Physical Sciences University of Newcastle Callaghan NSW Australia; ^2^ Radiation Oncology Department Calvary Mater Newcastle Newcastle NSW Australia; ^3^ School of Health Sciences Faculty of Health and Medicine University of Newcastle Callaghan NSW 2308 Australia; ^4^ Hunter Medical Research Institute (HMRI) Imaging Centre New Lambton Heights NSW Australia; ^5^ Radiology Department Calvary Mater Newcastle Newcastle NSW Australia; ^6^ CSIRO Australian e‐Health Research Centre Herston Queensland Australia; ^7^ School of Electrical Engineering and Computing University of Newcastle Callaghan NSW Australia

**Keywords:** machine learning, multiparametric MRI, probability map, prostate cancer, radiomics

## Abstract

**Purpose:**

The aim of this study was to develop and assess the performance of supervised machine learning technique to classify magnetic resonance imaging (MRI) voxels as cancerous or noncancerous using noncontrast multiparametric MRI (mp‐MRI), comprised of T2‐weighted imaging (T2WI), diffusion‐weighted imaging (DWI), and advanced diffusion tensor imaging (DTI) parameters.

**Materials and methods:**

In this work, 191 radiomic features were extracted from mp‐MRI from prostate cancer patients. A comprehensive set of support vector machine (SVM) models for T2WI and mp‐MRI (T2WI + DWI, T2WI + DTI, and T2WI + DWI + DTI) were developed based on novel Bayesian parameters optimization method and validated using leave‐one‐patient‐out approach to eliminate any possible overfitting. The diagnostic performance of each model was evaluated using the area under the receiver operating characteristic curve (AUROC). The average sensitivity, specificity, and accuracy of the models were evaluated using the test data set and the corresponding binary maps generated. Finally, the SVM plus sigmoid function of the models with the highest performance were used to produce cancer probability maps.

**Results:**

The T2WI + DWI + DTI models using the optimal feature subset achieved the best performance in prostate cancer detection, with the average AUROC , sensitivity, specificity, and accuracy of 0.93 ± 0.03, 0.85 ± 0.05, 0.82 ± 0.07, and 0.83 ± 0.04, respectively. The average diagnostic performance of T2WI + DTI models was slightly higher than T2WI + DWI models (+3.52%) using the optimal radiomic features.

**Conclusions:**

Combination of noncontrast mp‐MRI (T2WI, DWI, and DTI) features with the framework of a supervised classification technique and Bayesian optimization method are able to differentiate cancer from noncancer voxels with high accuracy and without administration of contrast agent. The addition of cancer probability maps provides additional functionality for image interpretation, lesion heterogeneity evaluation, and treatment management.

**Abbreviations**
AC: Attenuation Coefficient
ADC: Apparent Diffusion Coefficient
AUROC: Area Under Receiver Operating Characteristic curve
CFS: Correlation‐based Feature Selection
Cl: Linear Anisotropy
Cp: Planar Anisotropy
Cs: Spherical Anisotropy
CV: Coefficient of Variation
DCE: Dynamic Contrast Enhancement
DTI: Diffusion Tensor Imaging
DWI: Diffusion‐Weighted Imaging
FA: Fractional Anisotropy
GLCM: Gray‐Level Co‐occurrence Matrix
λ1: Axial diffusivity
MD: Mean Diffusivity
ML: Machine Learning
Mp‐MRI: Multiparametric Magnetic Resonance Imaging
PCa: Prostate Cancer
PI‐RADS: Prostate Imaging‐Reporting and Data System
PSA: Prostate‐Specific Antigen
PZ: Peripheral Zone
RA: Relative Anisotropy
RBF: Radial Basis Function
RD: Radial diffusivity
ROC: Receiver Operator Characteristic
ROI: Region Of Interest
SVM: Support Vector Machine
T2WI: T2‐Weighted Imaging
TRUS: Transrectal Ultrasound
VD: Volume Diffusivity

## 1 INTRODUCTION

Prostate cancer (PCa) is the most diagnosed cancer among men, and the second leading cause of cancer death in Australia.^1^ Multiparametric magnetic resonance imaging (mp‐MRI) is used as a complementary PCa diagnosis method to prostate‐specific antigen (PSA) test, digital rectal examination, and transrectal ultrasound (TRUS)‐guided biopsy for the detection and assessment of PCa.^2^, ^3^ Accurate spatial delineations of PCa lesions using mp‐MRI have many benefits. It can assist with treatment decisions and margins, enable focal treatments specifically targeting the lesion, or facilitate increases in radiotherapy doses (boosts) to the lesions. It may also play a role in evaluation of response to treatment. Mp‐MRI‐based delineation is made difficult by low contrast, potential discordances between the results of the different pulse sequences, and the similarities between the appearances of benign and malignant lesions. Recent advances of machine learning (ML) techniques have the potential to improve the efficiency, accuracy, and consistency of prostate mp‐MR delineations.^4^, ^5^ A combination of ML technique and human observer may provide the optimal accuracy for PCa delineation.^5^

According to the Prostate Imaging Reporting and Data System (PI‐RADS), the clinical guidelines for mp‐MRI include T2‐weighted imaging (T2WI), diffusion‐weighted imaging (DWI), and dynamic contrast enhancement (DCE).^6^ Several studies have investigated the potential of ML using T2WI, DWI, DCE, and diffusion tensor imaging (DTI).^7^, ^8^ DCE has a minor role in the PI‐RADS V2 guidelines, limited to confirming peripheral zone (PZ) DWI‐based PI‐RADS3 findings. While some studies have shown additional benefit for PZ,^9^ other studies have not shown a benefit including a recent study using a DTI sequence.^10^ DCE also requires a contrast agent adding to cost, time, and risk.^8^ DTI is an extension to DWI, which evaluates anisotropic water diffusion in tissue structures using different gradient directions. Previous studies confirmed the utility of DTI to evaluate normal and cancerous prostate anatomy employing the widely used parameters of fractional anisotropy (FA) and mean diffusivity (MD). DTI can also yield several other quantitative parameters that to our knowledge have not been evaluated in the prostate using ML, including axial diffusivity (AD) and volume ratio (VR).^11^ Therefore incorporation of DTI including these new parameters could improve PCa delineation with ML techniques.

The majority of previous prostate tumor delineation techniques have performed a binary cancer or noncancer voxel‐based classification. However producing a statistical probability of tumor presence may be useful for the noninvasive assessment of cancer heterogeneity and response to treatment. In addition, it may be useful to guide dose prescription for dose painting‐based treatment planning for PCa radiation therapy. Groenendaal et al. developed a logistic regression statistical model of the probability of tumor presence in PZ on a voxel level (2.5 mm^3^) using T2W, DWI, and DCE‐MRI.^12^ Development of more advanced ML models that derive tumor probability should therefore be beneficial to PCa diagnosis and treatment.

The aim of this study was to develop and assess the performance of supervised ML technique to classify voxels on noncontrast mp‐MRI as cancerous or noncancerous to delineate intermediate‐ and high‐risk biopsy‐proven disease of PZ. The method combines radiomic features extracted from T2WI and DWI incorporating advanced DTI parameters and develops a support vector machine (SVM) method to determine a voxel‐based cancer probability.

## 2 MATERIALS AND METHODS

### 2.A Patients

Seventeen men with high PSA and positive PCa biopsy were included in this study. All patients underwent 16‐core (S16C) 8‐zone transrectal and transperineal ultrasound‐guided biopsy at least 6 weeks before the MRI scan. All patients signed written consent prior to participating in this study, which was approved by the local health district human research ethics committee. One patient with Parkinson disease was excluded due to image distortion artifacts, leaving 16 patients. Clinical information of the patients is summarized in Table 1.

**Table 1:** Clinical information of the recruited peripheral zone (PZ) prostate cancer patients.

	Mean ± SD^a^	Range
Age(years)	70.21 ± 10.59	52–87
PSA (ng/mL)	18.92 ± 11.49	1.3–37.2

Biopsy Gleason score	Number of patients (total = 16)	%
3 + 4	3	18.7
4 + 3	6	37.5
4 + 5	5	31.2
5 + 4	2	12.5

^a^ SD, standard deviation.

The inclusion criteria were as follows: (a) patients with biopsy‐proven PCa with complete clinical data, (b) patients without any contraindication of using MRI and (c) at least 6 weeks after the biopsy. The exclusion criteria were as follows: (a) data with insufficient quality, (b) prior PCa treatment, (c) presence of hip prosthesis, (d) pelvic nodal involvement, and (e) men with intellectual impairment who would have difficulty giving informed consent to the study.

All patients underwent contrast‐free T2WI, DWI, and DTI. One main limitation of prostate diffusion MRI is the sensitivity to motion and prostate movement, such as air–tissue interfaces.^13^ In an attempt to overcome this limitation, all patients were restricted to a low‐fiber diet 24 hours before scan^14^ and were asked to empty bladder and received a rectal laxative (Microlax®, Sanofi‐Winthrop, Colombiers, France) before MRI sessions. These protocols showed a reduction in prostate gland shift in PCa patients treated with radiotherapy.^15^

### 2.B Image acquisition

Mp‐MRI examinations were carried out on a 3 tesla MRI scanner (Skyra, Siemens Healthineers, Germany) equipped with a phase array coil. The full mp‐MRI parameters are exhibited in Table 2. Mp‐MRI scans were obtained 6–8 weeks after prostate biopsy to avoid hemorrhage artifacts as suggested by PI‐RADS V2.^16^ Furthermore, T1‐weighted MRI scans were acquired for patients and bleeding artifacts were not observed.

**Table 2:** Multiparametric magnetic resonance imaging (mp‐MRI) sequence parameters used for this study.

	T2WI	DWI	DTI
Sequence	TSE	Single shot EPI	Single shot EPI
Echo time (ms)	96	65	101
Repetition time (ms)	1400	4600	11300
Flip angle (°)	135°	90°	90°
Field of view (mm^2^)	200 × 200	260 × 260	260 × 260
Slice thickness (mm)	4	4	4
Slice gap (mm)	0	0	0
Fat saturation	No	Yes	Yes
b‐values (s/mm^2^)	n/a	50, 400, 800	0, 1600
Voxel size (mm^2^)	0.8 × 0.8	2.0 × 2.0	1.7 × 1.7
Number of direction	n/a	3	30
Acquisition time (min)	3.45	4.19	6.62

T2WI: T2‐weighted imaging; DWI: diffusion‐weighted imaging; DTI: diffusion tensor imaging; TSE: turbo spin echo; EPI: echo planar imaging; min: minutes; n/a: not available.

### 2.C Image preprocessing

T2WI images were bias corrected using the N4ITK bias correction method with 3D Slicer software (www.Slicer.org) to eliminate intensity bias due to differing coil sensitivities. The N4ITK algorithm is based on nonparametric nonuniform intensity normalization (N3), with improvement in B‐spline smoothing strategy. The N4ITK optimization parameters included: B‐spline order of 3, B‐spline grid resolution of (1,1,1), a shrinkage factor of 4, a maximum number of 100 iterations at each of the three resolution levels, and a convergence threshold of 0.001. Noise reduction was then applied using a median filter with a 3 × 3 square window.^17^

To allow comparison between different patient T2WI intensities, four biological targets were considered for potential use as a reference for image standardization: femoral bone marrow, ischioanal fossa, obturator‐internus muscle, and urine. The biological reference reproducibility was assessed by the interpatient coefficient of variations (%interCV). The tissue with the highest reproducibility (lowest %interCV) was used for intensity standardization using median + interquartile intensity range method.^18^

For high b‐value DWI, noise reduction was applied using a diffusion anisotropic filter and bias corrected using the N4ITK bias correction method using 3D Slicer. Then, high b‐value DWI and apparent diffusion coefficient (ADC) maps were co‐registered with T2WI using a rigid and affine co‐registration method to correct motion misalignment and eddy current distortion in 3D Slicer.

The DTI volume was registered to b‐value = 0 s/mm^2^ with an affine and rigid body registration to correct for eddy current distortion artifacts and motion artifacts using ExploreDTI software (http://www.ExploreDTI.com).^19^ For noise reduction, an adaptive anisotropic diffusion filter was applied using the log‐Euclidean anisotropic filter available from the software package, MedINRIA.^20^ The filtered tensor images were imported into DTIStudio and used to generate eigenvalues (primary [$\lambda_{1}$], secondary [$\lambda_{2}$], and tertiary [$\lambda_{3}$]).^20^ Subsequently, these three eigenvalues served to calculate 20 DTI features using Matlab 2015b software (Mathworks, USA). Then, these maps were co‐registered with T2WI using a rigid and affine co‐registration method in 3D Slicer. All DWI and DTI upsampled to T2WI original matrix size.

### 2.D Lesion segmentation

**Fig. 1:**
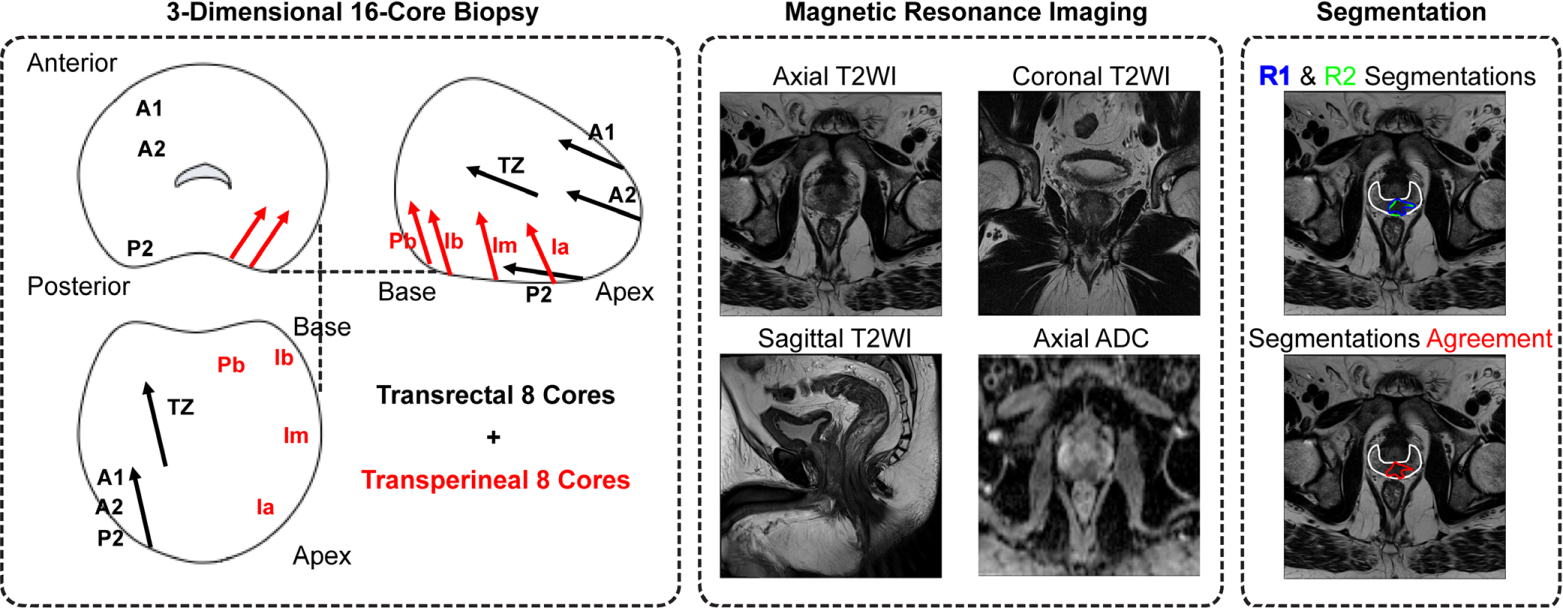
All patients underwent 16‐core (S16C) transrectal and transperineal ultrasound guided biopsy. Axial, coronal, and sagittal T2WI and axial DWI were acquired at least 6 weeks after biopsy. The two radiologists (R1 and R2) visually matched biopsy report and MRI. Then, cancer regions of interest (ROI)s were delineated by radiologists (blue and green colors). The agreement for each ROI between the two radiologists was calculated and used as cancer ROI (red color). Peripheral zone (PZ) boundaries (white color) were delineated by either radiologist.

All images were individually assessed by two experienced radiologists; one radiologist with more than 20 years and one with 12 years of experience in prostate radiology. In both cases, image assessment was performed in conjunction with the biopsy‐reported cancer location. Radiologists visually matched ADC map, baseline image (b‐value = 0 s/mm^2^), and corresponding T2WI slice locations and gland anatomy (apex, mid‐gland area, and base). The cancer regions of interest (ROI)s on separate multiple slices for each patient were manually outlined independently by each radiologist in PZ on T2WI using OsiriX software (OsiriX V.0.9.0, Pixmeo, Geneva, Switzerland). The cancer ROIs were in most cases on adjacent slices but not always and so a three‐dimensional cancer ROI was not constructed. PZ boundaries were delineated by either one of radiologists. The agreement for each cancer ROI between the two radiologists was assessed (Radiologist #1; n = 50 and Radiologist #2; n = 49) using the Dice similarity index. In order to maximize the identification accuracy of the cancer regions, only the overlap between cancer ROIs (n = 47) was used for training and testing.^21^ Radiologist #1 delineated a ROI with volume 0.14 cc that Radiologist #2 did not identify. Also there was no overlap between two ROIs with volume <0.25 cc selected by two radiologists. Therefore, these three ROIs were excluded from the study. The rest of PZ (i.e., the PZ excluding cancer ROIs) was then used as the noncancer ROIs. Figure 1 shows the scheme of the full prostate lesion segmentation process.

### 2.E Supervised machine learning

**Fig. 2:**
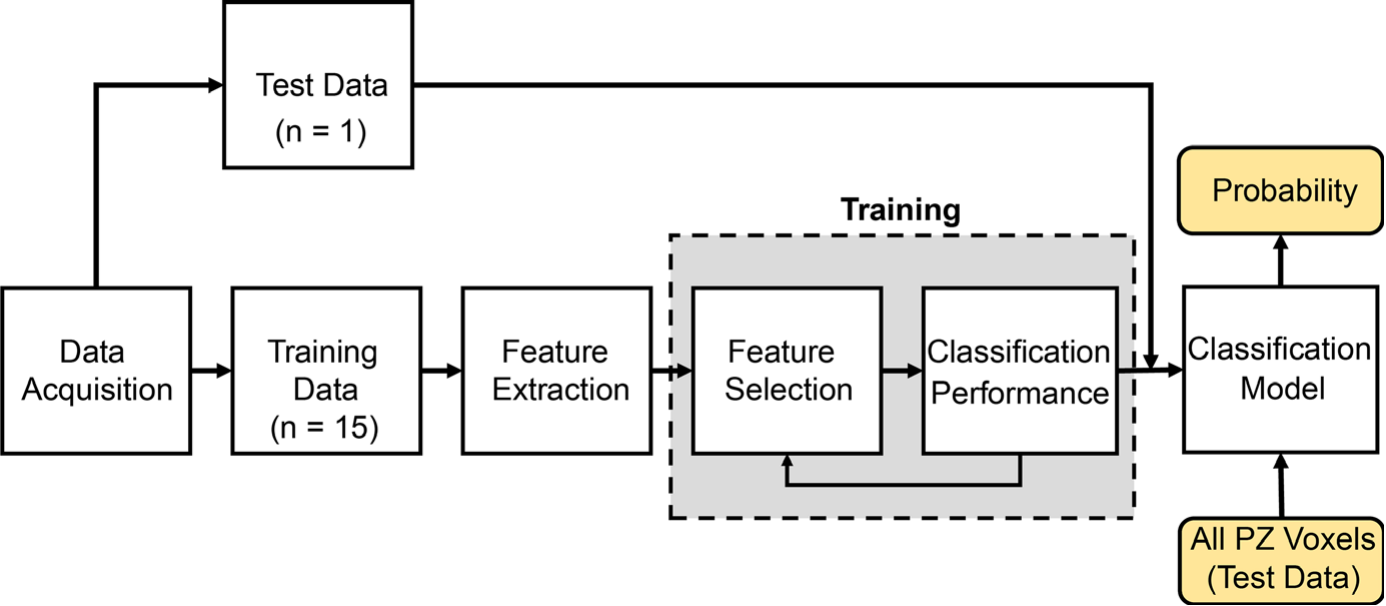
*General work flow diagram of the supervised machine learning system and probability map generation for peripheral zone (PZ).*

The voxel‐by‐voxel approach offers for a precise tumor analysis.^22^ In this study, voxel‐based analysis was used to develop a ML system for cancer prediction. Figure 2 shows a schematic diagram of the implemented in the current study.

#### 2.E.1 Radiomic feature extraction

In this study, 191 different features were extracted from each voxel within cancer and noncancer ROIs on mp‐MRI using Matlab 2015b software (Mathworks, USA). These quantitative features comprised 171 features from T2WI, high b‐value DWI and ADC map, and 20 diffusivity and anisotropy features from DTI.

Features were derived for each voxel of T2WI, high b‐value DWI, and ADC map according to the following five main categories:

1. Gray‐level features; image intensity of standardized T2WI, DWI, and ADC map.
2. Fifteen first‐order texture features: mean, 25th percentile, 75th percentile, variance, standard deviation, median, interquartile range, mode, minimum, maximum, range (maximum ‐ minimum), skewness, kurtosis, entropy, and energy; extracted over a sliding window.^23^ Texture feature evaluation requires rather large windows in order to obtain meaningful descriptions of their content. However, small windows are required in order to accurately locate the boundaries between different textured regions. In this study, a 9 × 9 voxel sliding window was used for texture features extraction.^24^
3. Nineteen second‐order texture features: variance, standard deviation, contrast, maximum probability, energy, entropy, correlation, maximum correlation coefficient, inertia, inverse, homogeneity, dissimilarity, cluster shade, sum of average, sum of variance, sum of square, sum of entropy, difference variance, and difference entropy; extracted over a 9 × 9 voxel sliding window using a gray‐level co‐occurrence matrix (GLCM).^25^
4. Six edge‐based features extracted to evaluate the local intensity variations; Roberts, Prewitt, Sobel, Canny, Roberts, and log.^26^
5. Sixteen gradient‐based features derived in three directions (horizontal‐x, vertical‐y, diagonal‐xy) and the magnitude was measured using Sobel, Prewitt, and Robert gradient operators and Central and Intermediate difference gradient methods.

Twenty quantitative diffusivity and anisotropy features were extracted from DTI volumes using tensor eigenvalues (λ_1_, λ_2_, and λ_3_).^11^ The most common prostate DTI measures are MD and FA. In this study, eight anisotropy parameters (FA, relative anisotropy (RA), volume ratio (VR), linear ($C_{l}$), planar ($C_{p}$) and spherical ($C_{s}$) anisotropies, attenuation coefficient (AC), and mode) and 12 diffusivity parameters (MD, axial diffusivity ($\lambda_{1}$), two orthogonal diffusivities to $\lambda_{1}\left(\lambda_{2}\;\text{and}\;\lambda_{3}\right)$ and four radial diffusivities (RD, $\lambda_{1}‐\lambda_{2}$, $\lambda_{1}‐\lambda_{3}$, and $\lambda_{2}‐\lambda_{3}$),^27^ in addition to novel DTI parameters, volume diffusivity (VD $=\lambda_{1}\lambda_{2}\lambda_{3}$), and surface diffusivities ($S_{e_{1}e_{2}}=\lambda_{1}\lambda_{2},\;S_{e_{1}e_{3}}=\lambda_{1}\lambda_{3}\;\text{and}\;S_{e_{2}e_{3}}=\lambda_{2}\lambda_{3}$) ) were extracted from DTI volumes.^11^

#### 2.E.2 Feature selection

The extracted voxel features from the different image volumes represent multidimensional feature vectors. However, some of these features are either irrelevant, weakly discriminating or are duplicative and therefore can be removed without loss of information. Feature selection is an important component of ML as it simplifies the classification model, decreases training time and enhances generalization by reducing overfitting. In this study, a correlation‐based feature selection (CFS) method was used to extract global features for each model, based on the premise that useful feature subsets contain features that are predictive of the class but uncorrelated with one another. The CFS method computes a heuristic measure of the “merit” of a feature subset from pairwise feature correlations and a formula adapted from test theory.^28^ The CFS method was applied to the training data set to find the optimal feature set. Before performing feature selection, each of the extracted features was normalized to be more sensitive to the classification model using the minimum–maximum scale method so that the minimum value was 0 and maximum value was 1.

#### 2.E.3 Classification and validation

The cancer voxels and noncancer voxels were utilized from all patients in the training data sets. Consider the training data set ${\left(x_{i},y_{i}\right)}_{i=1,\ldots ,n}$ of n input vectors of m features, where $x_{i}\in R^{m}$, i=1,.…,n, and $x_{i}=\left[x_{i,1},x_{i,2},\ldots ,x_{i,m}\right]$, along with their corresponding labels ${\left(y_{i}\right)}_{i=1,\ldots ,n}$, where $y_{i}\in \left\{‐1,+1\right\}$ for i=1,.…,n. For a linearly separable training data set, hard‐margin SVM is based on margin maximization between two classes and is defined as:

$$f_{\mathrm{lin}}\left(x\right)=〈x,w〉+b$$

where w is the normal vector to the hyperplane and b is the intercept of the decision boundary.

However, linear SVM is usually not successful in practice and nonlinear SVM is required.^29^ The training data set are represented as points and SVM transforms them into feature space by a nonlinear function ψ and the linear SVM is trained for the transformed samples ${\left\{\psi \left(x_{i}\right)\right\}}_{i=1}^{n}$.

$$f_{\mathrm{nonlin}}\left(x\right)=\left\langle \psi \left(x\right),w\right\rangle +b$$

In this study, a nonlinear SVM using a radial basis function (RBF) kernel (RBF‐SVM) $K\left(x,x_{i}\right)=\exp\left(‐\gamma \|x‐x_{i}\|\right)$ was used for classification. Bayesian optimization of SVM regularization parameter C and the Gaussian kernel shape parameter γ was used to find the optimal values within an interval of [10^−4^, 10^3^] using tenfold cross validation.^30^ The performance of the model for discrimination of cancer and noncancer voxels was measured using the area under receiver operating characteristic curve (AUROC). RBF‐SVMs were developed on the training data sets using the reduced features.

Overfitting is a common and serious problem. To reduce overfitting, a leave‐one‐patient‐out approach was used for model validation for assessing how the classification model will generalize an independent data set.^31^ Then, a comprehensive set (4 × 16) of different RBF‐SVM models (T2WI, T2WI + DWI, T2WI + DTI, and T2WI + DWI + DTI) were developed and optimized using the training data sets. For each model, the data set was divided into two parts, a training set (15 patients) and a test set (one patient). The test data sets were used to validate the optimized classification models and the average sensitivity, specificity, and accuracy of the models were measured. A nonparametric Mann–Whitney U‐test was applied to compare the diagnostic performance of different model sets using IBM SPSS statistic version 0.24.0 software (IBM Corp., Armonk, NY, USA) and *P* < 0.05 was considered to indicate statistical significance. Additionally, Dice similarity index between radiologists' agreement and output of models with the highest diagnostic performance were also calculated.

#### 2.E.4 Probability map

The output of the binary SVM model for input $x_{i}=\left[x_{i,1},x_{i,2},\ldots ,x_{i,m}\right]$ is a decision function $f\left(x\right)$ such that $sign\left(f\left(x\right)\right)$ belongs to one of two classes labels $\left\{‐1,+1\right\}$ and can be used to estimate the labels, cancer and noncancer. However if the model gives the degree of certainty about the label, then the output of the model can be described as a probability $\left(y_{i}|x_{i}\right)$ instead of a binary ‐1/1 label. A sigmoid function was fitted to the SVM decision function to derive the probability distribution:^32^

$$P\left(\left(y_{i}=1|x_{i}\right)\right)\approx P_{A,B}\left(f\right)\equiv \frac{1}{1+\exp\left(Af+B\right)},\text{where}f=f_{\mathrm{nonlin}}\left(x\right)$$

A and B are two scalar parameters that are learned by the algorithm. The parameters A and B are optimized by minimizing the local negative log‐likelihood:

$$‐\sum_{k=1}^{n}t_{k}\log\left(p_{k}\right)+\left(1‐t_{k}\right)\log\left(l‐p_{k}\right)$$

where, $p_{k}$ denotes the output of the sigmoid and $t_{k}$ the probability target. To solve this problem model‐trust minimization algorithm based on Newton’s method was used.

## 3 RESULTS

For T2WI intensity standardization, the urine demonstrated the least consistency between patients (%interCV = 35.7%), while ischioanal fossa provided the best overall consistency considering interpatient variability (%interCV = 10.6%). Thus, T2WI intensity standardization was performed by dividing the original signal intensity of T2WI by the median + interquartile range intensity of ischioanal fossa.

The overall Dice similarity index between the radiologist cancer ROIs was 0.78 ± 0.21. Two ROIs selected by two radiologists with volumes <0.5 cc and >0.25 cc had an average Dice similarity index of 0.54 ± 0.02 but the use of the overlap of the two radiologists improves the certainty in the cancer ROI as far as possible.

### 3.A Feature extraction and selection

**Fig. 3:**
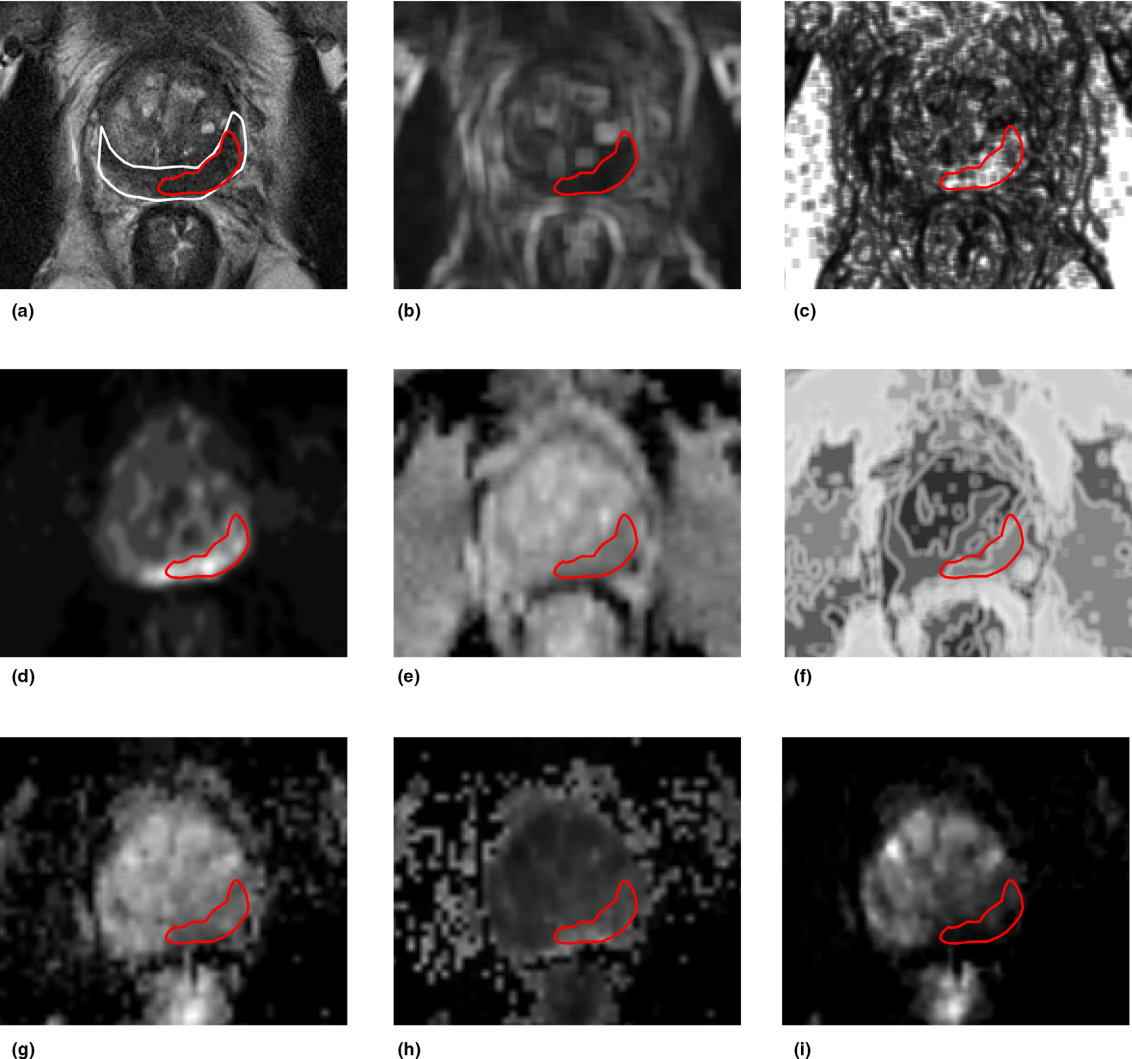
Examples of extracted features from multiparametric magnetic resonance imaging (mp‐MRI) from a 68‐yr‐old prostate cancer patient with serum prostate‐specific antigen (PSA) of 21.0 ng/ml, who was diagnosed with biopsy‐proven Gleason grade 4 + 3 in the left peripheral zone (PZ). Magnetic resonance imaging (a) axial T2WI with delineation of PZ (white) and cancer (red) regions of interest (ROI)s, (b) standard deviation of T2WI, (c) energy map of T2WI, (d) sum average of high b‐value DWI, (e) apparent diffusion coefficient (ADC) map, (f) sum entropy of ADC, (g) λ_3_, (h) attenuation coefficient (AC) of DTI and (i) volume diffusivity of DTI.

The total number of radiomic features calculated using T2WI, DWI, and DTI were 57, 114, and 20, respectively. Figure 3 shows some examples of extracted radiomic feature maps from T2WI, high b‐value DWI, and DTI.

Subsets of features were selected for each of the four different classification sets using a correlation‐based approach. The feature selection method also reports the relative importance of the selected features in terms of their predictive capability. Due to leave‐one‐patient‐out validation method, the number of selected features was different for different model sets. However, a number of common features were selected among all model sets. These features are called commonly selected features.

For the T2WI models the most common important features were the intensity of T2WI, first‐order texture features (standard deviation map and minimum) and second‐order texture features (cluster shade and sum entropy). Homogeneity and energy of second‐order texture features were important for only two of the T2WI models.

For the T2WI + DWI models, the ADC map from DWI had the highest predictive capability for the discrimination of the cancer and noncancer voxels. The most common effective additional features selected were T2WI signal intensity, T2WI first‐order texture feature (minimum) and T2WI second‐order texture feature (sum entropy), high b‐value DWI intensity; high b‐value DWI second‐order texture features (entropy and cluster shade), ADC second‐order texture features (sum variance, energy, and sum entropy), and ADC gradient‐based features (Roberts in diagonal direction). Other less frequent features selected by the T2WI + DWI models were second‐order texture features of T2WI (homogeneity and energy), first‐order texture feature of ADC (skewness), and edge‐based feature of ADC (Canny).

For the T2WI + DTI models, the signal intensity of T2WI, sum entropy of T2WI, diffusivity features extracted from DTI (VD,$\lambda_{1}‐\lambda_{2}$, AC, trace, and RD), and anisotropy features from DTI (FA) were commonly selected and had high predictive capability. Other less frequently selected features were second‐order texture features of T2WI (homogeneity and energy) and VR and RA features from DTI.

**Fig. 4:**
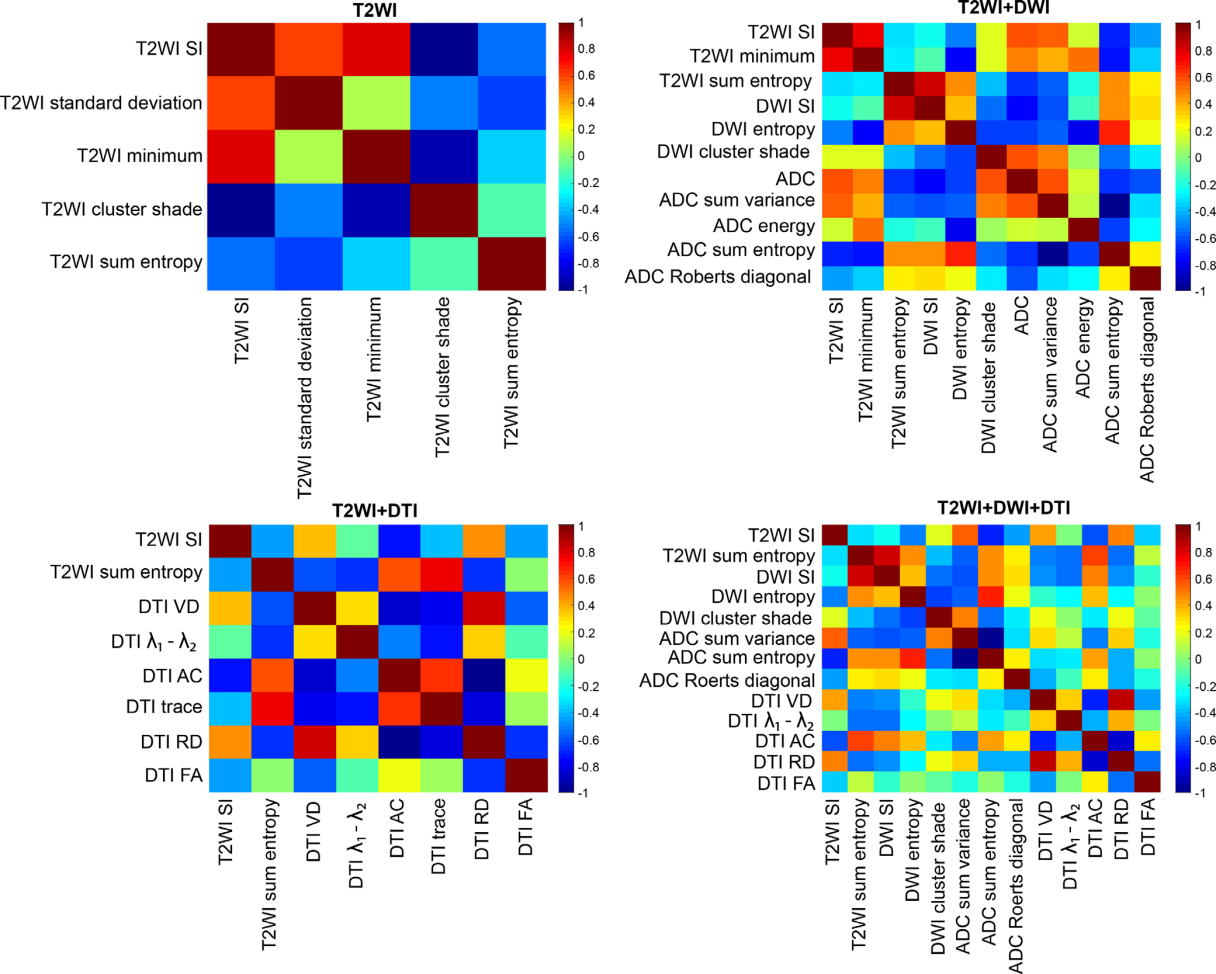
Pearson correlation heat maps of the commonly selected features for T2WI, T2WI + DWI, T2WI + DTI, and T2WI + DWI + DTI models from first‐order texture features, second‐order texture features, edge and gradient‐based features of T2WI, apparent diffusion coefficient (ADC) map, high b‐value DWI, and diffusivity and anisotropy feature of DTI with correlation score bar range [−1, 1] on the right side.

For the T2WI + DWI + DTI models, the volume diffusivity map from DTI had the most common effective predictive performance. The additional radiomic features were T2WI intensity, second‐order texture feature of T2WI (sum entropy), DWI intensity, second‐order features of high b‐value DWI (entropy and cluster shade), second‐order texture features of ADC (sum variance and sum entropy), gradient‐based feature of ADC map (Robert in diagonal direction), diffusivity feature of DTI (VD, $\lambda_{1}‐\lambda_{2}$, AC, and RD), and anisotropy feature of DTI (FA). Other less frequently selected features were second‐order texture features of T2WI (homogeneity and energy), first‐order texture feature of ADC (skewness), edged‐based feature of ADC (Canny), and VR and RA features from DTI. The Pearson correlation heat maps of the most common extracted features for T2WI, T2WI + DWI, T2WI + DTI, and T2WI + DWI + DTI models are displayed in Fig. 4. To generate the correlation coefficient maps, the commonly selected features at each voxel of ROI were summarized into mean value per ROI. Then the Pearson correlation coefficient of the mean values among ROIs for all data sets (training and test) were calculated and the corresponding maps generated.

### 3.B Classification and validation

**Fig. 5:**
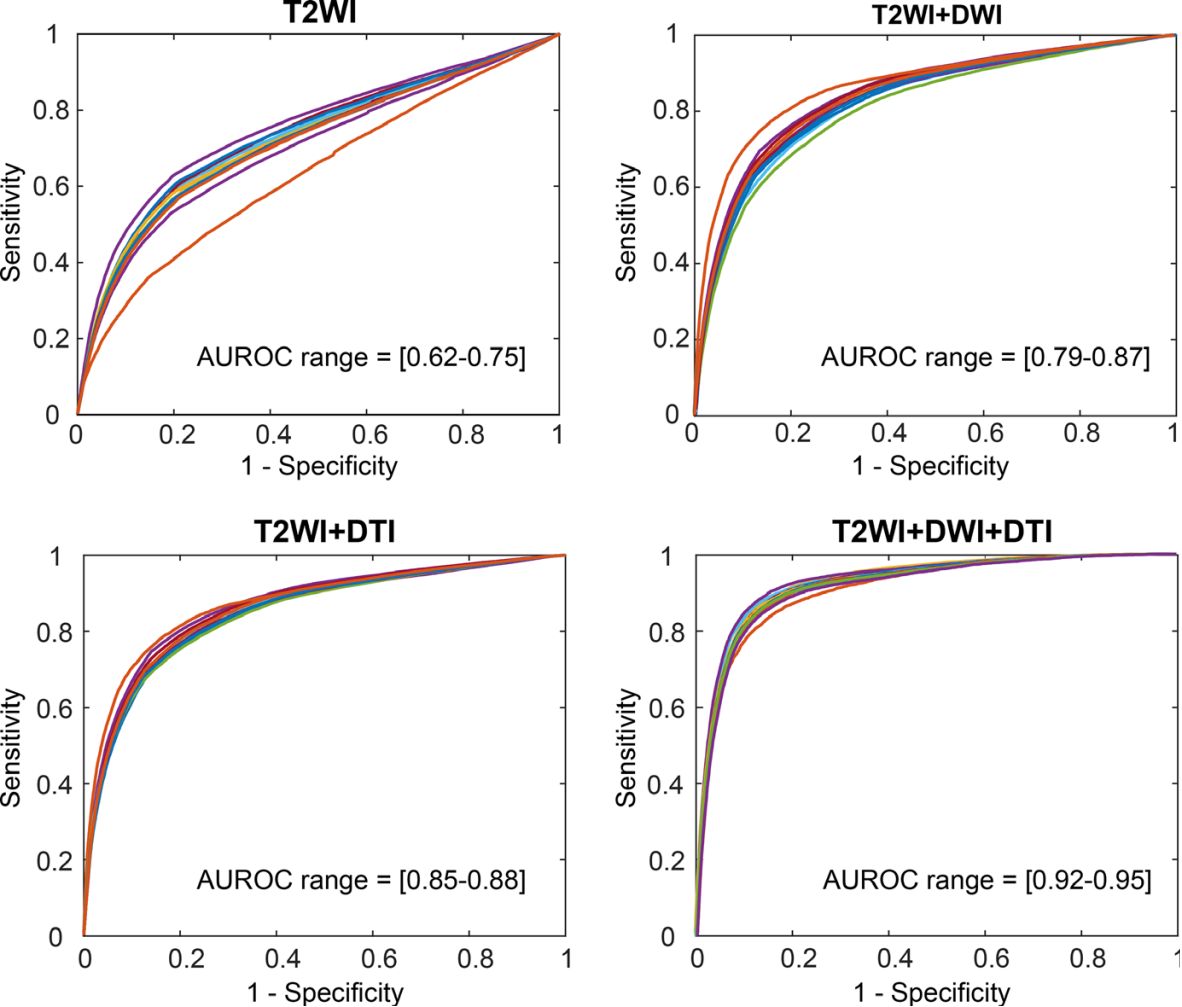
Support vector machine using a radial basis function (RBF‐SVM) classifications performance of T2WI, T2WI + DWI, T2WI + DTI, and T2WI + DWI + DTI using leave‐one‐patient‐out approach for the selected features when combined with correlation‐based feature selection (CFS) method. For each model and corresponding curve, the data set was divided into two parts, a training set (15 patients) and a test set (one patient) and a comprehensive set (4 × 16) of different RBF‐SVM models were generated.

**Fig. 6:**
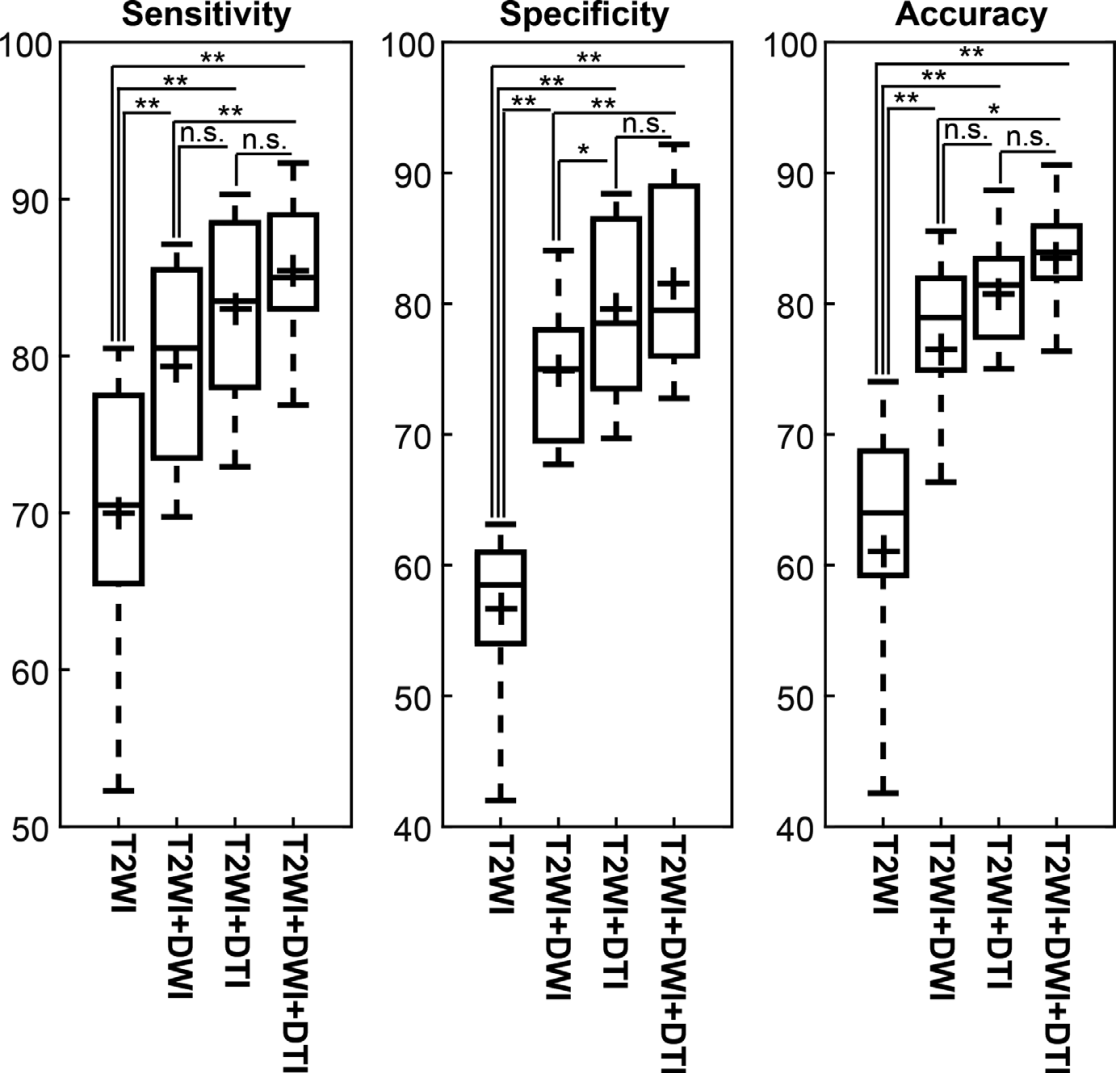
Boxplot comparing the sensitivity, specificity, and accuracy statistics of support vector machine using a radial basis function (RBF‐SVM) classifiers using leave‐one‐patient‐out validation approach. Mann–Whitney *U*‐test, **P < 0.01, *P < 0.05 and n.s. *P* > 0.05.

The kernel parameters of C and γ for each model with the minimum objective function value for a total of 30 for function evaluation iterations are summarized in Table 3. T2WI + DWI + DTI models achieved the largest value of γ, which indicates the more peaked the corresponding transformations of the feature vectors with the higher capacity of the models. The kernel parameters control the trade‐off between error due to bias and variance in the model. Using the optimal parameters, the RBF‐SVM models were developed and the receiver operating characteristic curves of the set of T2WI, T2WI + DWI, T2WI + DTI, and T2WI + DWI + DTI models using leave‐one‐patient‐out approach plotted for the discrimination of cancer and noncancer voxels (Fig. 5). For each model and corresponding curve plotted in Fig. 5, the data set was divided into two parts, a training set (15 patients) and a test set (one patient) and a comprehensive set (4 × 16) of different RBF‐SVM models were generated. The T2WI + DWI + DTI models using the optimal feature subset achieved significantly higher diagnostic performance using training data sets in PCa voxel discrimination, with the average AUROC of 0.93 ± 0.03, range [0.92–0.95] than T2WI, T2WI + DWI, and T2WI + DTI (*P* < 0.01). The trained models derived were used on the test data sets. The average AUROC, accuracy, sensitivity, and specificity of models using the test data sets are summarized in Table 3. The voxel‐based T2WI + DWI + DTI models detect cancer voxels in PZ with the average sensitivity, specificity, and accuracy of 0.85 + 0.05, 0.82 + 0.07, and 0.83 + 0.04, respectively. Our results demonstrated that adding DWI and/or DTI to T2WI can significantly improve the sensitivity, specificity, and accuracy of PCa identification in PZ compare to T2WI alone (*P* < 0.01). Furthermore, combination of T2WI, DWI, and DTI can significantly improve the sensitivity, specificity, and accuracy of PCa compare to T2WI + DWI (*P* < 0.05). The specificity of T2WI + DTI model was significantly higher than T2WI + DWI (*P* < 0.05). However, there was no significant difference in sensitivity and accuracy between T2WI + DWI and T2WI + DTI models (*P* > 0.05). Figure 6 shows boxplots of sensitivity, specificity, and accuracy of SVM models using test data sets. The average Dice similarity index of the segmentation using T2WI + DWI + DTI models on a voxel level by comparing the predicted segmentation with the radiologist agreement was 0.82 ± 0.16, range: 0.69–0.92.

**Table 3:** The most commonly selected and average of optimal kernel parameters for T2WI, T2WI + DWI, T2WI + DTI, and T2WI + DWI + DTI models and estimated AUROC, sensitivity, specificity, and accuracy results ± standard deviation of a comprehensive set of support vector machine using a radial basis function (RBF‐SVM) models.

	T2WI	T2WI + DWI	T2WI + DTI	T2WI + DWI + DTI
Commonly selected features/total features	5/57	11/114	8/77	13/191
AUROC	0.72 ± 0.02	0.83 ± 0.02	0.86 ± 0.07	0.93 ± 0.03
C(×10^−4^)	3.52 ± 0.07	2.21 ± 0.04	2.28 ± 0.05	1.02 ± 0.02
γ	291.32 ± 8.25	321.14 ± 11.27	303.89 ± 15.57	350.38 ± 112.30
Sensitivity (%)	0.70 ± 0.11	0.79 ± 0.07	0.83 ± 0.07	0.85 ± 0.05
Specificity (%)	0.56 ± 0.09	0.75 ± 0.06	0.80 ± 0.08	0.82 ± 0.07
Accuracy (%)	0.61 ± 0.10	0.77 ± 0.08	0.81 ± 0.05	0.83 ± 0.04

AUROC, area under receiver operating characteristic curve; CI, confidence interval.

### 3.C Binary and probability maps

**Fig. 7:**
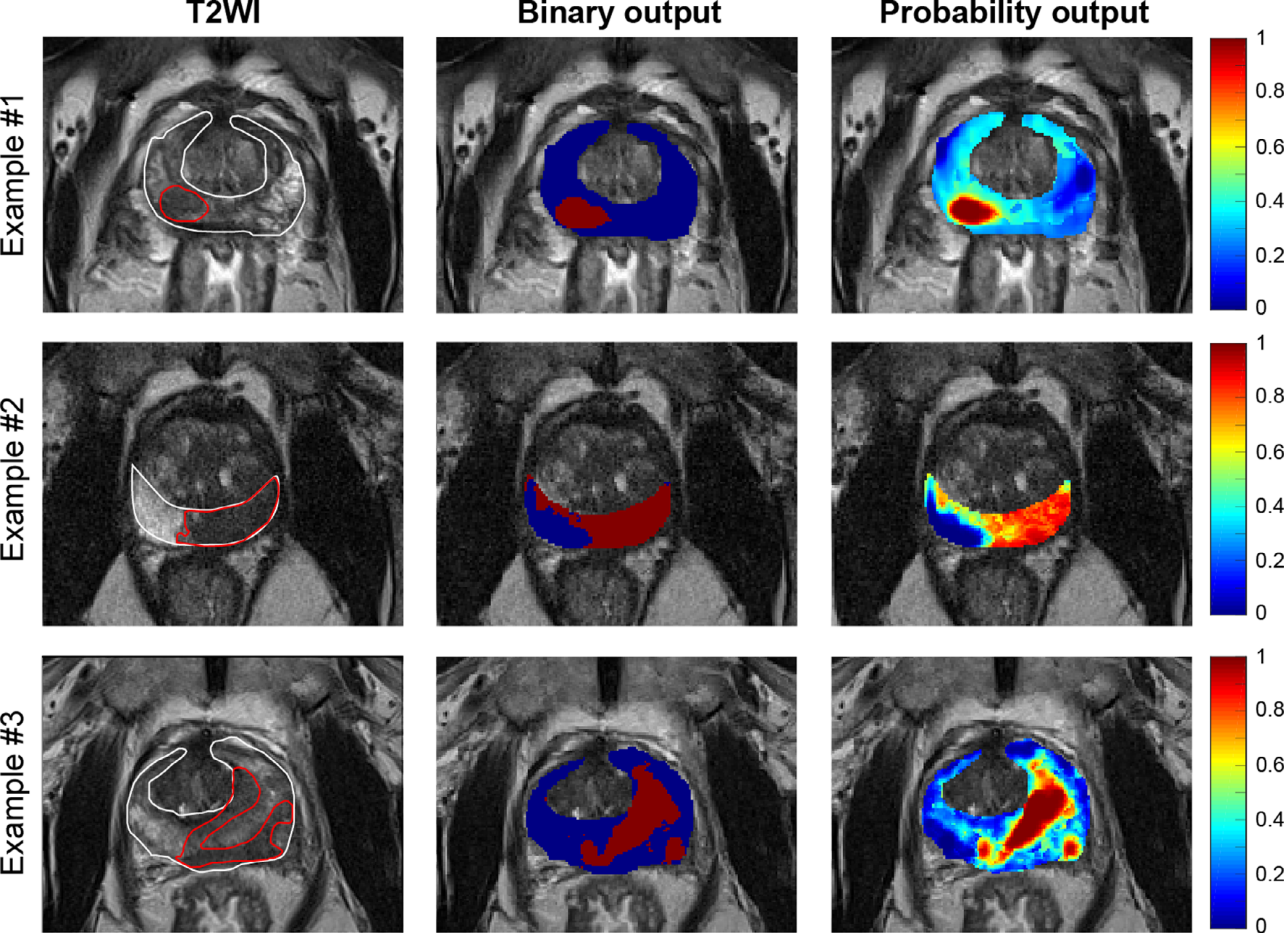
Examples of T2WI, binary map, and probability map for three prostate cancer patients with Gleason score of 4 + 5, 4 + 3, and 3 + 4, respectively. Delineations of peripheral zone (PZ) (white) and cancer (red) regions of interest (ROI) overlay on T2WI were performed by concordance of two experience radiologists.

The T2WI + DWI + DTI models were used to generate binary and probability outputs of different models in the entire PZ of test data sets. Figure 7 shows the T2WI, binary output, and posterior probability of the T2WI + DWI + DTI RBF‐SVM models for PZ for three patients with different Gleason scores. In addition, the overlap between two radiologist segmentations of the tumor on T2WI figures allows us to visually assess the efficacy of the models.

First example of Fig. 7 is a 70‐yr‐old patient with serum PSA of 11.60 ng/ml who was diagnosed with biopsy‐proven 4 + 5 Gleason grade in the right PZ. AUROC of this model for differentiating cancer from noncancer voxels using the training data set was 0.95. This model successfully identified the dominant lesion with sensitivity and spasticity of 0.87 and 0.90, respectively, on the test patient. Second example is a 68‐yr‐old PCa patient with biopsy‐proven Gleason grade 4 + 3 in the left PZ (PSA of 21.0 ng/ml). The corresponding AUROC of RBF‐SVM model using the training data set was 0.94. The sensitivity and specificity of the second model on the test patient were 0.93 and 0.78, respectively. Third example is a PCa in an 81‐yr‐old PCa patient with serum PSA of 1.27 ng/ml, who was diagnosed with biopsy‐proven 3 + 4 Gleason grade in the left PZ. AUROC of the corresponding RBF‐SVM model was 0.92. For this patient, although the probability map demonstrated the high probability of abnormality in the area depicted by the radiologists, the model did not include all the voxels inside the cancer ROI. This model identified the cancer lesion with sensitivity and specificity of 0.71 and 0.83, respectively.

## 4 DISCUSSION AND CONCLUSION

In the current study, we developed multiple voxel‐based ML models using an RBF‐SVM classification algorithm and Bayesian optimization method for detection of PCa in PZ. We have shown that our ML technique with an RBF‐SVM and CFS approach can identify most of the radiologist reported PCa voxels in PZ. The average AUROC for the combination of T2WI, DWI, and DTI was 0.93 ± 0.03 for discriminating cancer and noncancer voxels. Our results are comparable to the performance of previously reported studies for voxel‐based ML of PCa using mp‐MRI in either PZ using different classification algorithms, such as SVM or linear discrimination analysis.^5^, ^33^ Nevertheless, due to different data sets and factors used in previous studies, it is difficult to make comparisons between results. In this study, we only considered cancer to be present if identified by both radiologists; hence only voxels within the radiologist overlap regions were considered cancer. In total, 47 cancer regions were identified by both radiologists. T2WI + DWI + DTI models positively identified cancer voxels within all cancer ROIs or lesions. In that sense, all lesions were detected. Using the voxel results the positive results within the radiologist lesions had an average sensitivity of 0.85 ± 0.05 (i.e., on average of 85% of the voxels within the lesion were identified as cancer — true positives). The results would be used clinically as visual guidance to the presence of lesions. However, for voxel‐based methods if individual voxel results used to denote lesion presence there will be a very high false‐positive number. Therefore, evaluating the diagnostic performance of voxel‐based ML based on the false‐positive detected areas is not reliable enough due to the areas giving rise to individual false‐positive voxel.^34^ Such a diagnostic technique may allow for faster patient evaluations while helping to increase diagnostic consistency. This study demonstrated the feasibility of voxel‐based ML systems using contrast‐free MR and the achieved diagnostic performance was consistent with the other studies. DTI provides additional information about microstructure of the prostate tissue due to the restricted movement of water molecules in tissues. Mp‐MRI using DTI achieved better diagnostic performance for PCa in PZ without the potential side effects of DCE‐MRI from injection and long acquisition time.^24^, ^35^, ^36^ There has been an increasing interest in utilizing high b‐value diffusion MRI to improve the specificity of high‐grade PZ PCa detection despite their lower signal‐to‐noise ratio.^37^ In this study, a b‐value of 1600 s/mm^2^ was used to improve specificity of cancer detection. We acquired high‐quality prostate DTI in 30 different gradient directions to improve signal‐to‐noise ratio without endorectal coil.^38^, ^39^ Most prostate DTI studies emphasized MD and FA parameters in cancer and noncancer tissues.^40^, ^41^ In this study, multiple DTI maps were generated to evaluate more structured or packed organization of cancer cells and/or tissue.^42^ This study also investigated four additional quantitative DTI parameters for PCa detection, namely volume diffusivity and three surface diffusivity maps. In this study we found that sum entropy of T2WI have the potential to differentiate between PCa and noncancerous prostate voxels for T2WI, T2WI + DWI, T2WI + DTI, and T2WI + DWI + DTI models. Sum entropy provides the texture pattern of inhomogeneity inside the tumors.

The ADC map from DWI had the highest predictive capability for the discrimination of the cancer and noncancer voxels for T2WI + DWI models. ADC is a measure of the magnitude of diffusion of water molecules within tissue. Due to cellularity of cancer tissue, this magnitude is significantly different between cancer and noncancer tissues.

For T2WI + DTI and T2WI + DWI + DTI models, diffusivity parameters of DTI (e.g., VD) demonstrated highly successful discrimination between cancer and noncancer voxels. Diffusivity parameters of DTI calculate the magnitude of diffusion of water molecules in multiple different direction (at least six directions) while anisotropy features describes the degree of anisotropy of a diffusion process.

This study showed that Haralick‐based texture features over a 9 × 9 sliding window extracted from T2WI, DWI, and ADC map of DWI can improve differentiation between cancer and noncancer tissue.^43^ There is no single optimum window size at which the best result can be achieved. However, a small window size such as 3 × 3 does not provide sufficient information about limited intensities and gray‐level variation and using a large window size (e.g., 15 × 15) increase the chance of mixing up benign and malignant voxels. Experimental results suggested median window size of 9 × 9 as the best window size for prostate texture feature on MRI.^24^, ^44^, ^45^

Overfitting with small sample size is a common problem in ML.^31^ To eliminate biased estimation, the training and test data sets were separated for model validation using a leave‐one‐patient‐out approach, all features normalized using the min–max scale method, features selected from training pool using a leave‐one‐patient‐out approach, and model parameters optimized using Bayesian optimization technique. These models successfully predict unseen test data set with high performance, thus highlighting the non‐overfitting properties of classifiers.

Quantitative evaluation of the diagnostic performance of different classification algorithms for prostate mp‐MRI demonstrated that SVM yields the maximum AUROC and the highest accuracy.^24^ Although mostly used for binary classification, SVM can be adapted to calculate probabilities. The probability map produced in this study can be used as a useful tool to evaluate tumor heterogeneity and response to treatment.

The low number of patients is a limitation of this study, and the results can only be considered preliminary at this stage. Studying a cohort with more widely spread Gleason scores, supported by standard anatomic specimens, is warranted. Another limitation of this study is that classification and validation were based on radiologists' determination rather than section histology. Radiologist reporting performance cannot be perfect, and some errors are inevitable. However, this has been offset by only considering intermediate and high‐grade cancer in this study and by using the concordance of two expert radiologists to minimize this uncertainty. In addition, cancer ROIs were selected as the overlap between two radiologists' delineation, and noncancer ROIs were selected as the rest of PZ. However, noncancer ROIs might contain cancerous voxels, which is another limitation of the current study.

In conclusion, we have developed two classes of discrimination: a binary (cancer vs noncancer) and a probability RBF‐SVM in this study. Binary output of SVM makes it impossible to distinguish lesion heterogeneity. A combined supervised ML technique and radiomic approach appeared as a feasible tool to predict cancer lesion on noncontrast mp‐MRI. The probability output of the models provide useful practical cancer recognition information for malignancy risk assessment. The binary classification is a subclass of the RBF‐SVM probability models. This probability map conveys a human readable result that the radiologist may use during patient evaluation. False‐positive results in binary and probability maps remain a challenge. The Bayesian optimization method was used to minimize the false‐positive diagnosis on the binary and probability maps.

## CONFLICT OF INTEREST

The authors declare no conflict of interest.

5

## References

[1] Ferlay J , Shin HR , Bray F , et al. Estimates of worldwide burden of cancer in 2008: GLOBOCAN 2008. Int J Cancer. 2010;127:2893–2917. 21351269 10.1002/ijc.25516

[2] Barry MJ . Prostate‐specific–antigen testing for early diagnosis of prostate cancer. New Engl J Med. 2001;344:1373–1377. 11333995 10.1056/NEJM200105033441806

[3] Hodge KK , McNeal JE , Terris MK , et al. Random systematic versus directed ultrasound guided transrectal core biopsies of the prostate. J Urol. 1989;142:71–74. 2659827 10.1016/s0022-5347(17)38664-0

[4] Wernick MN , Yang Y , Brankov JG , et al. Machine learning in medical imaging. IEEE Sign Process Mag. 2010;27:25–38. 10.1109/MSP.2010.936730 PMC4220564 25382956

[5] Lemaître G , Martí R , Freixenet J , et al. Computer‐aided detection and diagnosis for prostate cancer based on mono and multi‐parametric MRI: a review. Comput Biol Med. 2015;60:8–31. 25747341 10.1016/j.compbiomed.2015.02.009

[6] Turkbey B , Choyke PL . PIRADS 2.0: what is new? Diagn Interv Radio. 2015;21:382. 10.5152/dir.2015.15099 PMC4557320 26200484

[7] Hamoen EH , de Rooij M , Witjes JA , et al. Use of the prostate imaging reporting and data system (PI‐RADS) for prostate cancer detection with multiparametric magnetic resonance imaging: a diagnostic meta‐analysis. Eur Urol. 2015;67:1112–1121. 25466942 10.1016/j.eururo.2014.10.033

[8] Moradi M , Salcudean SE , Chang SD , et al. Multiparametric MRI maps for detection and grading of dominant prostate tumors. J Mag Reson Imaging. 2012;35:1403–1413. 10.1002/jmri.23540 PMC5478377 22267089

[9] Greer MD , Shih JH , Lay N , et al. Validation of the dominant sequence paradigm and role of dynamic contrast‐enhanced imaging in PI‐RADS version 2. Radiology. 2017;285:859–869. 28727501 10.1148/radiol.2017161316 PMC5708285

[10] Kozlowski P , Chang SD , Jones EC , et al. Assessment of the need for DCE MRI in the detection of dominant lesions in the whole gland: correlation between histology and MRI of prostate cancer. NMR Biomed. 2018;31:e3882. 10.1002/nbm.3882 29266527

[11] Gholizadeh N , Greer PB , Simpson J , et al. Characterization of prostate cancer using diffusion tensor imaging: a new perspective. Eur J Radiol. 2019;110:112–120. 30599846 10.1016/j.ejrad.2018.11.026

[12] Groenendaal G , Borren A , Moman MR , et al. Pathologic validation of a model based on diffusion‐weighted imaging and dynamic contrast‐enhanced magnetic resonance imaging for tumor delineation in the prostate peripheral zone. Int J Radiat Oncol Biol Phys. 2012;82:e537–e544. 22197085 10.1016/j.ijrobp.2011.07.021

[13] Charles‐Edwards EM , Nandita MDJCI . Diffusion‐weighted magnetic resonance imaging and its application to cancer. Cancer Imaging. 2006;6:135–143. 17015238 10.1102/1470-7330.2006.0021 PMC1693785

[14] McNair HA , Wedlake L , McVey GP , et al. Can diet combined with treatment scheduling achieve consistency of rectal filling in patients receiving radiotherapy to the prostate? Radiother Oncol. 2011;101:471–478. 21903283 10.1016/j.radonc.2011.08.003

[15] Yahya S , Zarkar A , Southgate E , et al. Which bowel preparation is best? Comparison of a high‐fibre diet leaflet, daily microenema and no preparation in prostate cancer patients treated with radical radiotherapy to assess the effect on planned target volume shifts due to rectal distension. Br J Radiol. 2013;86:20130457. 23995876 10.1259/bjr.20130457 PMC3830438

[16] Barrett T , Turkbey B , Choyke PL . PI‐RADS version 2: what you need to know. Clin Radiol. 2015;70:1165–1176. 26231470 10.1016/j.crad.2015.06.093 PMC6369533

[17] Tustison NJ , Avants BB , Cook PA , et al. N4ITK: improved N3 bias correction. IEEE Trans Med Imaging. 2010;29:1310–1320. 20378467 10.1109/TMI.2010.2046908 PMC3071855

[18] Gholizadeh N , Fuangrod T , Greer PB , et al. An inter‐centre statistical scale standardisation for quantitatively evaluating prostate tissue on T2‐weighted MRI. Australas Phys Eng Sci Med. 2019;42:137–147. 30637607 10.1007/s13246-019-00720-1

[19] Soares J , Marques P , Alves V , et al. A hitchhiker's guide to diffusion tensor imaging. Front Neurosci. 2013;7:31. 23486659 10.3389/fnins.2013.00031 PMC3594764

[20] Sinha S , Sinha U , Malis V , et al. Exploration of male urethral sphincter complex using diffusion tensor imaging (DTI)‐based fiber‐tracking. J Magn Reson Imaging. 2018;48:1002–1011. 29573022 10.1002/jmri.26017 PMC6151300

[21] Vinod SK , Min M , Jameson MG , et al. A review of interventions to reduce inter‐observer variability in volume delineation in radiation oncology. J Med Imaging Radiat Oncol. 2016;60:393–406. 27170216 10.1111/1754-9485.12462

[22] Wang S , Burtt K , Turkbey B , et al. Computer aided‐diagnosis of prostate cancer on multiparametric MRI: a technical review of current research. Biomed Res Int. 2014;2014:789561. 25525604 10.1155/2014/789561 PMC4267002

[23] Kyriacou E , Nicolaides A , Pattichis CS , et al. First and second order statistical texture features in carotid plaque image analysis: preliminary results from ongoing research. Conf Proc IEEE Eng Med Biol Soc. 2011;2011:6655–6658. 10.1109/IEMBS.2011.6091641 22255865

[24] Niaf E , Rouvière O , Mège‐Lechevallier F , et al. Computer‐aided diagnosis of prostate cancer in the peripheral zone using multiparametric MRI. Phys Med Biol. 2012;57:3833. 22640958 10.1088/0031-9155/57/12/3833

[25] Davnall F , Yip CSP , Ljungqvist G , et al. Assessment of tumor heterogeneity: an emerging imaging tool for clinical practice? Insights into Imaging. 2012;3:573–589. 23093486 10.1007/s13244-012-0196-6 PMC3505569

[26] Zhou XS , Huang TS . Edge‐based structural features for content‐based image retrieval. Pattern Recognit Lett. 2001;22:457–468.

[27] Van Hecke W , Emsell L , Sunaert S . Diffusion Tensor Imaging: A Practical Handbook. Berlin: Springer; 2015.

[28] Hall MA . Correlation‐based feature selection of discrete and numeric class machine learning; 2000.

[29] Cortes C , Vapnik V . Support‐vector networks. Mach Lear. 1995;20:273–297.

[30] Snoek J , Larochelle H , Adams RP . Practical Bayesian optimization of machine learning algorithms. Adv Neural Inf Process Syst. 2012;2951–2959.

[31] Vabalas A , Gowen E , Poliakoff E , et al. Machine learning algorithm validation with a limited sample size. PLoS One. 2019;14:e0224365. 31697686 10.1371/journal.pone.0224365 PMC6837442

[32] Platt J . Probabilistic outputs for support vector machines and comparisons to regularized likelihood methods. Advan Large Margin class. 1999;10:61–74.

[33] Gholizadeh N , Pundavela J , Nagarajan R , et al. Nuclear magnetic resonance spectroscopy of human body fluids and in vivo magnetic resonance spectroscopy: potential role in the diagnosis and management of prostate cancer. Urol Oncol. 2020;38:150–173. 31937423 10.1016/j.urolonc.2019.10.019

[34] Suzuki K . Pixel‐based machine learning in medical imaging. J Biomed Imaging. 2012;2012:1. 10.1155/2012/792079 PMC3299341 22481907

[35] Ampeliotis D , Antonakoudi A , Berberidis K , et al. ICSPC 2007. IEEE Int Conf Signal Process Commun. 2007;2007:888–891.

[36] Puech P , Betrouni N , Makni N , et al. Computer‐assisted diagnosis of prostate cancer using DCE‐MRI data: design, implementation and preliminary results. Int J Comput Ass Rad Sur. 2009;4:1–10. 10.1007/s11548-008-0261-2 20033597

[37] Agarwal HK , Mertan FV , Sankineni S , et al. Optimal high b‐value for diffusion weighted MRI in diagnosing high risk prostate cancers in the peripheral zone. J Magn Reson Imaging. 2017;45:125–131. 27383502 10.1002/jmri.25353 PMC6364696

[38] Shenhar C , Degani H , Bar Y , et al. Innovative diffusion MRI protocol to improve prostate cancer diagnosis. Eur Urol Suppl. 2018;17:e1883–e1884.

[39] Zhan L , Chiang M‐C , Barysheva M , et al. How many gradients are sufficient in high‐angular resolution diffusion imaging (HARDI). *Workshop Comput Diffus MRI, MICCAI*; 2008:216–224.

[40] Gibbs P , Pickles MD , Turnbull LW . Diffusion imaging of the prostate at 3.0 tesla. Invest Radiol. 2006;41:185–188. 16428991 10.1097/01.rli.0000192418.30684.14

[41] Sinha S , Sinha U . In vivo diffusion tensor imaging of the human prostate. Magn Reson Med. 2004;52:530–537. 15334571 10.1002/mrm.20190

[42] Westin CF , Maier SE , Mamata H , et al. Processing and visualization for diffusion tensor MRI. Med Image Ana. 2002;6:93–108. 10.1016/s1361-8415(02)00053-1 12044998

[43] Avanzo M , Stancanello J , El Naqa I . Beyond imaging: The promise of radiomics. Phys Med. 2017;38:122–139. 28595812 10.1016/j.ejmp.2017.05.071

[44] Rampun A , Wang L , Malcolm P , et al. A quantatiitive study of texture features across different window sizes in prostate T2‐weighted MRI. Procedia Comput Sci. 2016;90:74–79.

[45] Chen M , Dang HD , Wang JY , et al. Prostate cancer detection: comparison of T2‐weighted imaging, diffusion‐weighted imaging, proton magnetic resonance spectroscopic imaging, and the three techniques combined. Acta Radiol. 2008;49:602–610. 18568549 10.1080/02841850802004983

